# Excitability of the Motor Cortex in *De Novo* Patients with Celiac Disease

**DOI:** 10.1371/journal.pone.0102790

**Published:** 2014-07-25

**Authors:** Giovanni Pennisi, Giuseppe Lanza, Salvatore Giuffrida, Luisa Vinciguerra, Valentina Puglisi, Mariagiovanna Cantone, Manuela Pennisi, Carmela Cinzia D'Agate, Pietro Naso, Giuseppe Aprile, Giulia Malaguarnera, Raffaele Ferri, Rita Bella

**Affiliations:** 1 Department “G.F. Ingrassia”, Section of Neurosciences, University of Catania, Catania, Italy; 2 Department of Neurology I.C., Oasi Institute for Research on Mental Retardation and Brain Aging (I.R.C.C.S.), Troina (Enna), Italy; 3 Department of Chemistry, University of Catania, Catania, Italy; 4 Gastroenterology and Endoscopy Unit, University of Catania, Catania, Italy; 5 Department of Biomedical Sciences, University of Catania, Catania, Italy; University of Ottawa, Canada

## Abstract

**Introduction:**

Celiac disease (CD) may initially present as a neurological disorder or may be complicated by neurological changes. To date, neurophysiological studies aiming to an objective evaluation of the potential central nervous system involvement in CD are lacking.

**Objective:**

To assess the profile of cortical excitability to Transcranial Magnetic Stimulation (TMS) in a group of *de novo* CD patients.

**Materials and methods:**

Twenty CD patients underwent a screening for cognitive and neuropsychiatric symptoms by means of the Mini Mental State Examination and the Structured Clinical Interview for DSM-IV Axis I Disorders, respectively. Instrumental exams, including electroencephalography and brain computed tomography, were also performed. Cortico-spinal excitability was assessed by means of single and paired-pulse TMS using the first dorsal interosseus muscle of the dominant hand. TMS measures consisted of resting motor threshold, motor evoked potentials, cortical silent period (CSP), intracortical inhibition (ICI) and facilitation (ICF). None of the CD was on gluten-free diet. A group of 20 age-matched healthy controls was used for comparisons.

**Results:**

CD showed a significantly shorter CSP (78.0 *vs* 125.0 ms, *p*<0.025), a reduced ICI (0.3 *vs* 0.2, *p*<0.045) and an enhanced ICF (1.1 *vs* 0.7, *p*<0.042) compared to controls. A dysthymic disorder was identified in five patients. The effect size between dysthymic and non-dysthymic CD patients indicated a low probability of interference with the CSP (Cohen's *d* -0.414), ICI (-0.278) and ICF (-0.292) measurements.

**Conclusion:**

A pattern of cortical excitability characterized by “disinhibition” and “hyperfacilitation” was found in CD patients. Immune system dysregulation might play a central role in triggering changes of the motor cortex excitability.

## Introduction

Celiac disease (CD) is a systemic autoimmune disorder triggered by gliadin ingestion in genetically susceptible individuals [1]–[3]. Genetic factors strongly contribute to CD, especially regarding HLA-DQ2 and DQ8, but also involving other non-HLA regions [1], [4], [5].

Although the main target in CD is the proximal small bowel, the clinical presentation is highly heterogeneous, ranging from asymptomatic to dramatically symptomatic forms and affecting several organs, such as skin, joints, bones, blood cells, endocrine glands, the reproductive system and the nervous system. In this context, approximately 50% of CD patients manifest extraintestinal dysfunctions and up to 22.5% have otherwise unexplained neurological symptoms [6]. Furthermore, neurological disorders may complicate the course or represent the onset of CD. Ataxia, peripheral neuropathy and seizures (with or without cerebral calcifications) are the most common neurological complications, with cerebellar ataxia being the most frequent, often associated with cerebellar atrophy at neuroimaging [7]. A possible association between CD and progressive cognitive impairment can be particularly observed, at neuropsychological tests evaluating verbal memory and executive functions [8], [9]. Different neuropsychiatric disorders, such as schizophrenia, depression, and anxiety, have also been reported to be associated with CD [10]. Therefore, CD should be considered in patients with unexplained neurological disorders and a neurological screening might disclose valuable information in these patients.

Nevertheless, although there is a relevant impact of CD and its complications on health and social life, to date techniques allowing an objective evaluation of the potential central nervous system involvement in CD are lacking.

In the last years, several investigators have used transcranial magnetic stimulation (TMS) to define the electrophysiological profile of several neuropsychiatric disorders [11], [12], physiological brain aging [13], different models of cognitive decline [14], [15] and some systemic diseases with a neurological involvement [16], [17]. TMS is a safe and non-invasive neurophysiological technique specifically able to evaluate the excitability and functioning of the primary motor cortex and the cortico-spinal tract [18]. TMS studies have provided findings that, although not disease specific, shed light on the cortical pathophysiology and the neurochemical basis underlying disease processes and represent a rationale for the plasticity-based interventions [18], [19]. More recently, TMS-derived parameters have allowed to explore the regulatory mechanisms of cortical excitability, supporting the concept of a cortical motor network whose output is also influenced by non-primary motor areas, including ventral and dorsal premotor cortex, supplementary motor area and cingulate cortex [20]. In particular, it is known that the cingulate cortex, together with the dorsolateral prefrontal cortex, is crucial for cognition and mood regulation [21].

Converging evidences suggest that gluten-mediated immune response in CD is associated with neuropsychiatric manifestations. Given that glutamic acid decarboxylase (GAD) antibodies may interfere with the GABAergic synaptic transmission, thus affecting inhibitory interneurons activity, in the present paper we first aimed to explore the potential involvement of inhibitory and facilitatory intracortical circuits to single- and paired-pulse TMS in *de novo* CD patients. We hypothesized that gluten intolerance might be associated with changes of specific TMS measures of excitation and inhibition.

## Materials and methods

### Ethics Statement

The study was approved by the ethics committee of the Azienda Ospedaliero-Universitaria “Policlinico-Vittorio Emanuele”, Catania (Italy). Written informed consent was obtained from all participants prior to participation in accordance with the Declaration of Helsinki. All assessments were performed in a controlled laboratory environment.

### Subjects

Twenty *de novo* CD patients (4 males and 16 females; median age 33.0 years, interquartile range 24.0–45.0), according to the European Society for Paediatric Gastroenterology Hepatology and Nutrition (ESPGHAN) guidelines for the diagnosis of CD [22], were consecutively recruited from the Regional Center for Celiac Disease of the University of Catania, Italy. The clinical-serological features and the main findings from the diagnostic work-up of CD patients are summarized in Table 1. Twenty age-matched healthy volunteers (8 males and 14 females; median age 29.5 years, interquartile range 26.0–45.5) were used as a control group. At the time of the examination, none of the patients was on gluten-free diet. Exclusion criteria were: major neurological disorder (i.e. Parkinson's disease, stroke, Alzheimer's disease, etc.); head trauma or epilepsy; acute, chronic or not compensated medical illness (i.e. myocardial infarction, kidney or liver failure, heart failure, etc.); Mini Mental State Examination score <24 [23], alcohol or drug abuse; age <18 years; use of drugs affecting cortical excitability (i.e. mood stabilizers, benzodiazepines, antipsychotics); any condition precluding TMS execution. On a total sample of 23 consecutive CD patients, 2 were excluded because of current intake of benzodiazepines and one refused the TMS protocol.

**Table 1:** Clinical-serological features and diagnostic work-up of CD patients.

Patient	Symptoms at onset	Co-morbidities	Antibodies	Endoscopy	Histopathology
1	Dyspepsia, tiredness	-	tTG, EMA	Scalloped duodenal folds	3b
2	Tiredness	-	tTG, EMA	Reduced duodenal folds	3b
3	Abdominal pain	Thyroiditis	tTG, EMA	Scalloped duodenal folds	3b
4	Diarrhea, abdominal pain	-	tTG, EMA	Reduced duodenal folds	3c
5	Constipation	-	tTG	Mucosal fissures	3b
6	Diarrhea	Asthma	tTG, EMA	Mosaic pattern	3c
7	Poliabortivity, tiredness	-	tTG, EMA	Mucosal fissures	3c
8	Abdominal pain	Thyroiditis	tTG, EMA	Reduced duodenal folds	3c
9	Dyspepsia, tiredness	Thyroiditis	tTG, EMA	Mosaic pattern	3c
10	None (screening, family history for CD)	-	tTG	Mosaic pattern	3c
11	Diarrhea, abdominal pain	Asthma	tTG, EMA	Absent duodenal folds	3c
12	Weight loss	Thyroiditis	tTG, EMA	Mosaic pattern	3c
13	Abdominal pain	-	tTG	Mucosal fissures	3b
14	Weight loss, diarrhea	Vitiligo	tTG, EMA	Scalloped duodenal folds	3a
15	Infertility, tiredness	Thyroiditis	tTG, EMA	Reduced duodenal folds	3c
16	Diarrhea	-	negative	Scalloped duodenal folds	3a
17	Weight loss	-	tTG, EMA	Mosaic pattern	3c
18	Weight loss, abdominal pain,	-	tTG, EMA	Mosaic pattern	3c
19	Dyspepsia, abdominal pain, diarrhea	-	tTG, EMA	Mosaic pattern	3c
20	Tiredness	Thyroiditis	tTG, EMA	Mosaic pattern	3c

CD  =  celiac disease; tTG  =  tissue transglutaminase antibodies; EMA  =  endomysial antibodies.

Histopathological classification is based on the Marsh–Oberhuber grading system [51]. 3a: mild villous flattening; 3b: marked villous flattening; 3c: total villous flattening.

### Assessment

The clinical-demographic evaluation included: age, gender, education, handedness, social and living conditions, general and neurological examinations, and co-morbidities. Neuropsychological tests included a screening of overall cognitive functions (Mini Mental State Examination), evaluation of neuropsychiatric symptoms diagnosed by the Structured Clinical Interview for DSM-IV Axis I Disorders (SCID-I) and the 17-items Hamilton Depression Rating Scale [24], for the quantification of any depressive symptom. Cognitive assessment was performed by a physician blind to the aim of the study. Instrumental exams included standard electroencephalogram (EEG), brain computed tomography (CT) scan and both single- and paired-pulse TMS.

EEG was recorded by means of a Micromed Brain Quick (System Plus), with a standard montage according to the 10-20 International System and with a pre-cabled EEG head cap. Brain CT was acquired with a helical 64-slices General Electric Scanning, with 2.5 mm slice thickness. Only the clinical, neuropsychological and TMS studies were carried out in controls.

### Transcranial magnetic stimulation

TMS was performed using a High-power Magstim 200 magnetic stimulator (Magstim Co., Whitland, Dyfed, UK). A 70 mm figure-of-eight coil was held over the motor cortex at the optimum scalp position to elicit Motor Evoked Potentials (MEPs) in the contralateral First Dorsal Interosseous (FDI) muscle of the dominant hand, according to the Edinburgh Handedness Inventory [25]. Resting motor threshold (rMT) was defined as the lowest stimulus intensity able to elicit MEPs at rest of an amplitude >50 µV in at least 5 of 10 trials, according to the IFCN recommendation [26]. Central motor conduction time was calculated by subtracting the conduction time in peripheral nerves, estimated by F wave techniques, from MEP latency obtained during moderate active muscle contraction, with a stimulus intensity set at 130% of the rMT. M and F waves are elicited by giving supramaximal electrical stimulation to the ulnar nerve at wrist. The size of the MEPs was expressed as a percentage of supramaximal M wave amplitude (A ratio). The cortical silent period (CSP) was determined with an approximately 50% of maximum tonic voluntary contraction of the FDI muscles, induced by single TMS pulses delivered at 130% of rMT. The mean CSP duration of 10 rectified trials was calculated. Intracortical inhibition (ICI) and Intracortical facilitation (ICF) were studied using the conditioning-test paradigm applying two magnetic stimuli in rapid succession [27]. The conditioning stimulus was set at 80% of the subjects rMT whereas the test stimulus at 130% of the rMT. The interstimulus intervals (ISIs) tested were 2, 3, 10 and 15 ms. Ten trials for each ISI were recorded in a random way with an 8-s interval between each trial. The responses were expressed as the ratio between the MEP amplitude produced by paired stimulation and that produced by TS alone. Paired-pulse TMS curves of intracortical excitability were obtained with a 70-mm figure-of-eight coil deriving pulses from a couple of Magstim 200 Stimulators, connected each other through a BiStim module (The Magstim Company, Whitland, Dyfed). The BiStim was connected to a CED Micro 1401 interface (Cambridge Electronic Design, Cambridge, UK) allowing stimulus generation and data capture. Electromyographic (EMG) activity was recorded with silver/silver-chloride disposable self-adhesive and self-conductive surface electrodes. The active electrode was placed over the muscular belly of the target muscle (FDI), the reference distally at the metacarpo-phalangeal joint of the index finger and the ground on the dorsal face of the wrist. For the motor nerve conduction study (M and F waves from the FDI muscle), a bipolar nerve stimulation electrode with 6-mm diameter felt pads and an interelectrode separation of 25 mm was used and applied to the ulnar nerve at wrist, bilaterally. All measurements were conducted while subjects were seated in a comfortable chair with continuous EMG monitoring to ensure either a constant level of EMG activity during tonic contraction or complete relaxation at rest. Data were collected and stored on a computer with an *ad-hoc* software, allowing data acquisition, processing and analysis [28]. To minimize the inter-subjects variability, all TMS procedures were performed in the same laboratory and situation, by the same operators and at the same time during the day.

### Statistical analysis

The comparison of the frequency of observation of some features in the CD group and in the control group was carried out by means of the Chi-square test. Because of the relatively low number of subjects in both groups and the non normal distribution of data (as determined by the Shapiro-Wilk's test of normality), all the other comparisons were performed by means of the non-parametric Mann-Whitney test for unpaired datasets. Differences were considered significant when they were below the *p*<0.05 level. However, because of the relatively limited number of subjects available and to rule out possible type II errors, we also calculated effect sizes using the Cohen's d value [29]. Cohen's *d* is defined as the difference between two means divided by their pooled standard deviation. According to Cohen, 0.2 is indicative of a small effect, 0.5 of a medium and 0.8 of a large effect size.

## Results

Demographic, clinical and neuropsychological characteristics of the participants are summarized in Table 2. The general examination of the CD group was unremarkable except for one overweight patient. The neurological examination of all patients was essentially normal. Nine patients had autoimmune co-morbidities, the most common being positive antithyroid peroxidase autoantibodies (six, altough euthyroid), followed by asthma (two) and vitiligo (one). Patients and controls were similar in terms of age, gender, handedness and educational level. The 17 item-HDRS scores were significantly worse in patients (7.0 *vs* 2.0, *p*<0.0058), whereas the SCID-I disclosed a dysthymic disorder in five CD patients; anxiety was also found in two of them. EEG and CT scan ruled out epileptic changes as well as intracranial calcifications or other clear neuroradiological abnormalities.

**Table 2:** Clinical features of the subjects included in this study.

	Celiac disease (n = 20)		Controls (n = 20)		Mann-Whitney		Effect size
	median	(25^th^-75^th^ quartile)	median	(25^th^-75^th^ quartile)	U	p<	Cohen's d
**Age, years**	33.0	(24.0–45.0)	29.5	(26.0–45.5)	180	NS	−0.123
**Education, years**	13.0	(9.0–13.0)	14.0	(13.5–14.0)	133	NS	−0.402
**MMSE**	30.0	(29.0–30.0)	30.0	(28.8–30.0)	182	NS	0.183
**17 item-HDRS**	7.0	(2.0–9.0)	2.0	(0.0–4.0)	98	0.0058	1.451
					**Chi^2^**	**p<**	
**Sex, males/females**	4/16		8/12		1.90	NS	
**Handedness, right/left**	18/2		18/2		0.00	NS	
**Neurological signs, yes/no**	3/17		0/20		3.24	NS	
**Comorbidity, yes/no**	9/11		0/20		11.60	0.001	
**SCID-I, yes/no**	5/15		0/20		5.71	0.017	

MMSE  =  Mini Mental State Examination; HDRS  =  Hamilton Depression Rating Scale; SCID-I  =  Structured Clinical Interview for DSM-IV Axis I.

**Figure 1:**
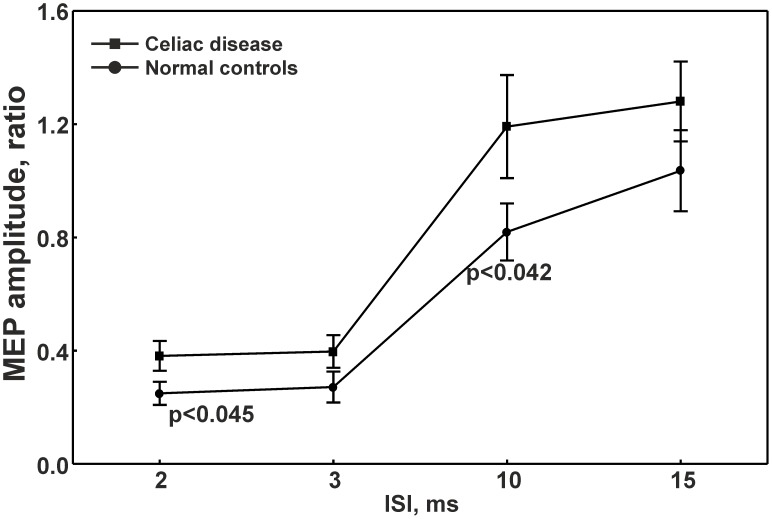
Intracortical excitability at different interstimuls intervals obtained from patients and controls. ISI  =  interstimuls interval; MEP  =  Motor Evoked Potential.

As shown in Table 3, CSP duration was significantly shorter in CD subjects with respect to controls (78.0 *vs* 125.0 ms, *p*<0.025), whereas no statistically significant differences were found for rMT. Fig. 1 shows curves of intracortical excitability at the different ISIs obtained in the two groups of participants. Conditioned MEPs amplitudes at ISI of 2 (0.3 *vs* 0.2, *p*<0.045) and 10 ms (1.1 *vs* 0.7, *p*<0.042) were significantly higher in CD patients than controls, indicating a reduced ICI and enhanced ICF in celiac individuals, respectively.

**Table 3:** Comparison of electrophysiological data in patients with celiac disease and controls.

	Celiac disease (n = 20)		Controls (n = 20)		Mann-Whitney		Effect size
	median	(25^th^-75^th^ quartile)	median	(25^th^-75^th^ quartile)	U	p<	Cohen's d
**rMT**	35.0	(34.0–41.5)	37.0	(32.0–40.0)	194.5	NS	0.070
**CSP, ms**	78.0	(61.5–100.0)	125.0	(69.5–160.0)	117	0.025	−0.872
**MEP latency, ms**	18.6	(18.2–19.7)	19.3	(18.5–20.2)	151	NS	−0.428
**CMCT**	5.9	(4.5–6.3)	5.8	(5.6–6.6)	168	NS	−0.368
**CMCTF**	4.8	(4.5–5.6)	5.3	(4.2–5.7)	175	NS	−0.148
**A ratio**	0.2	(0.2–0.5)	0.5	(0.3–0.7)	88.5	0.0026	−0.653
**F amplitude, µV**	0.1	(0.1–0.2)	0.1	(0.1–0.2)	179.5	NS	0.627

rMT  =  resting motor threshold; CSP  =  cortical silent period; MEP  =  Motor Evoked Potentials; CMCT  =  central motor conduction time; CMCTF  =  central motor conduction time estimated by F wave; A ratio  =  amplitude ratio.

Additionally, in order to evaluate the eventual influence of the five dysthymic CD patients on the TMS data, we have estimated the effect size between dysthymic and non-dysthymic CD patients, which was very low in all instances indicating a low probability of interference with the CSP (Cohen's *d* -0.414), ICI (-0.278) and ICF (-0.292) measurements.

## Discussion

This is the first multi-disciplinary investigation examining the impact of CD on motor cortex excitability. The main finding is the observation of electrophysiological changes within the motor cortex of these patients. In particular, we observed a significant reduction of the intracortical inhibitory amount indicated by shortened CSP and reduced ICI, together with a significant enhancement of ICF, in patients with CD compared to controls. This pattern, basically characterized by a “disinhibition” and “hyperfacilitation”, was observed in the absence of substantial changes of rMT. On the other hand, changes of different TMS measures without modification of rMT has already been reported and is likely due to the different electrophysiological and neurochemical basis that these measures have and explore [30], [31]. Resting MT represents a global parameter of cortical excitability and reflects the neuronal membrane excitability, as well as the local density of the excitatory interneurons and the cortico-spinal neurons within the motor cortex [18]. CSP, defined as the interval of suppressed voluntary EMG activity following a single-pulse TMS stimulus, is an index of motor cortical inhibition, basically due to the activation of GABA-B cortical inhibitory interneurons [32], [33]. ICI is probably mediated by GABA-A receptors [34], whereas ICF may represent an activating phenomenon arising from intracortical glutamatergic neurons [18], [35], but also tempered by GABAergic inhibition and modulated by serotoninergic, adrenergic, cholinergic and dopaminergic neurotransmission [36].

The mechanisms underlying this cortical disinhibition in CD patients are rather complex to explain, also because of the lack of similar studies. The pathophysiology of CD and the most accepted hypothesis explaining its neurological involvement may help understanding our results. Vitamins and trace elements deficiency is unlikely to explain the subtle neurophysiological changes to TMS, because it usually occurs in the most affected CD patients, in whom a wide intestinal damage is already evident, resulting in severe malabsorption and related consequences [7], [37].

An autoimmune-mediated pathogenesis for CD is supported by increasingly convincing evidence. Molecular mimicry between gliadin and nervous system proteins could lead to a cross-reaction of anti-gliadin antibodies (Abs) with nervous system antigens. Abs can react with Purkinje cells and peripheral nerve epitopes [10], [38]. Furthermore, anti-gliadin Abs have a immunoreactivity to synapsin I, which is very similar to gliadin because of the high frequency of proline and glutamine regions. Synapsin I is a neuronal phosphoprotein of the central and peripheral nervous system involved in forming and maintaining the reserve pool of synaptic vesicles and in managing neurotransmitter release [39], [40]. In CD patients, anti-gliadin Abs might possibly interfere with the normal balance between excitatory and inhibitory neural circuits as a consequence of the interaction with synapsin I.

Another interesting hypothesis regards a possible involvement of GABA, which is the main inhibitory neurotransmitter synthesized in the central nervous system from glutamate by GAD. Since GABA and GAD are also synthesized by neurons of the enteric plexus [41], anti-GAD Abs may arise in CD patients and concentrate in the Purkinje cells and peripheral nerves [42], [43] and may interfere with GABAergic synaptic transmission, thus affecting inhibitory interneurons activity [10].

Within humoral autoimmunity to neuronal antigens, anti-transglutaminase 2- or transglutaminase 6-related immunoglobulin depositions have been identified not only in the gut but also in the cerebellum, pons, medulla and around brain blood vessels [38], [44]. Additional findings show diffuse T-lymphocytic infiltration within the perivascular cuffing with inflammatory cells that could possibly damage the blood-brain barrier and expose the cerebral tissues to Abs [7], driving altered ion levels. Taken together, this may lead to a vicious circle resulting in a imbalance between inhibitory and facilitatory neuronal excitability.

Finally, growing evidence indicates that immune system dysregulation might play a central role in triggering neurological impairment in CD, leading not only to the neurophysiological alterations but also to neurobehavioral changes. In this context, another finding of our study is the evidence of a degree of depressive and anxiety symptoms in CD patients, adding further support to the relationship between psychiatric disorders and CD [45], [46]. Major depression has been widely studied by TMS, which has confirmed the key role of the GABAergic dysfunction in the neurochemical pattern of depressed mood, as indexed by a reduction of both CSP and ICI [11], [47]-[50]. In this view, it can be argued that our TMS findings might be explained as a result of depressive mood in five of the CD patients. However, to our knowledge no study assessing cortical excitability to TMS in dysthimia is available. Besides, in this series of patients, TMS modifications do not seem to be significantly influenced by the depressive disorder. Therefore, it can be hypothesized that the observed TMS changes of inhibitory circuits might be related to the synaptic disruption due to the gluten-mediated immune response and that an imbalance between inhibitory and facilitatory intracortical circuits occurs even at a subclinical neurological stage of CD.

This study has some limitations. First, TMS-related measures of cortical excitability do not provide specific pathophysiological information although they are sensitive to the “global weight” of several neurotransmitters, as well as to subcortical and cortical motor inputs. Second, the observed findings obtained from a relatively small number of patients do not allow to draw conclusions on the eventual causal relationship between CD and a specific pattern of cortical excitability to TMS. Therefore, these results need to be confirmed by further independent investigations with larger group sizes and to be correlated to the clinical presentation and course of CD, even after an adequate course of gluten-free dietary regimen. In this context, a “normalization” of the TMS parameters after the gluten-free diet might add support to the hypothesis of a direct correlation between TMS profile and CD.

In conclusion, this study reveals that CD patients seem to exhibit a relatively distinct pattern of cortical excitability to TMS, suggesting that even asymptomatic patients might disclose a subclinical neurological involvement. The identification of potential neuromarkers might be useful in the diagnosis, follow-up and prognosis of these patients, such as in the monitoring of any change of clinical, neuropsychological and neurophysiological data over time or after a gluten-free dietary regimen. TMS, together with clinical-cognitive, immunological and imaging data, can be considered to be an additional tool able to capture subtle changes in the pathophysiological and neurochemical mechanisms underlying the exciting connections between gut and brain.
